# Estimating coefficients for certain subclasses of meromorphic and bi-univalent functions

**DOI:** 10.1186/s13660-018-1879-4

**Published:** 2018-10-19

**Authors:** F. Müge Sakar

**Affiliations:** Department of Business Administration, Faculty of Management and Economics, Batman University, Batman, Turkey

**Keywords:** 30C45, 30C50, Univalent function, Bi-univalent function, Meromorphic function, Taylor–Maclaurin coefficients

## Abstract

In the present paper, we introduce two interesting subclasses of meromorphic and bi-univalent functions defined on $\Delta=\{z:z\in\mathbb{C}, 1<|z|<\infty\}$. For functions belonging to these subclasses, estimates on the initial coefficient $|b_{0}|$ and $|b_{1}|$ are obtained. Some other closely related results are also represented. The coefficient bounds presented here are new in their own kind. We hope that this paper will generate further interest in applying our approach to other related problems.

## Introduction and lemmas

Let $\mathcal{A}$ be the class of functions of the form

1.1 $$ f(z)=z+\sum_{k=2}^{\infty}a_{k}z^{k}, $$

which are analytic in the open unit disk $\mathbb{U}=\{z\in\mathbb{C}: |z|<1\}$. We also denote by $\mathcal{S}$ the subclass of the normalized analytic function class $\mathcal{A}$ consisting of all functions in which are also univalent in $\mathbb{U}$.

Since univalent functions are one-to-one, they are invertible and the inverse functions need not be defined on the entire unit disk $\mathbb{U}$. In fact, the Koebe one-quarter theorem [3] ensures that the image of $\mathbb{U}$ under every univalent function $f\in\mathcal{S}$ contains a disk of radius $1/4$. Thus every function $f\in\mathcal{S}$ has an inverse $f^{-1}$, which is defined by

$$ f^{-1}\bigl(f(z)\bigr)=z\quad(z\in\mathbb{U}), $$

and

$$ f \bigl(f^{-1}(w) \bigr)=w \quad \biggl( \vert w \vert < r_{0}(f); r_{0}(f)\geq \frac{1}{4} \biggr). $$

A function $f\in\mathcal{A}$ is said to be bi-univalent in the open unit disk $\mathbb{U}$ if both the function and its inverse are univalent in $\mathbb{U}$. Let *σ* denote the class of analytic and bi-univalent functions in $\mathbb{U}$ given by the Taylor–Maclaurin series expansion as in (1.1). For a brief history and interesting examples of functions in the class *σ*, see [19]. In fact, the aforecited work of Srivastava et al. [19] essentially revived the investigation of various subclasses of the bi-univalent function class *σ* in recent years; it was followed by works of, e.g., Frasin and Aouf [6], Srivastava et al. [18, 20], Xu et al. [21, 22] and others (see, for example, [1, 2, 4, 7, 11, 14]).

In this paper, the concept of bi-univalency is extended to the class of meromorphic functions defined on $\Delta=\{z:z\in\mathbb{C}, 1<|z|<\infty\}$. The class of functions

1.2 $$ g(z)=z+\sum_{k=0}^{\infty} \frac{b_{k}}{z^{k}} $$

that are meromorphic and univalent in Δ is denoted by Σ, and every univalent function *g* has an inverse $g^{-1}$ satisfying the series expansion

1.3 $$ g^{-1}(w)=w+\sum_{k=0}^{\infty} \frac{B_{k}}{z^{k}}, $$

where $0< M<|w|<\infty$. Analogous to the bi-univalent analytic functions, a function $g\in\Sigma$ is said to be meromorphic and bi-univalent if both *g* and $g^{-1}$ are meromorphic and univalent in Δ given by (1.2). The class of all meromorphic and bi-univalent functions is denoted by $\Sigma_{\sigma}$. A simple calculation shows that

1.4 $$ h(w)=g^{-1}(w)=w-b_{0}-\frac{b_{1}}{w}- \frac{b_{2}+b_{0}b_{1}}{w^{2}}-\frac{b_{3}+2b_{0}b_{2}+b_{0}^{2}b_{1}+b_{1}^{2}}{w^{3}}+\cdots. $$

Estimates on the coefficients of the inverses of meromorphic univalent functions were widely investigated in the literature. For example, Schiffer [15] showed that if *g*, defined by (1.2), is in Σ with $b_{0}=0$, then $|b_{2}|\leq2/3$. In 1971, Duren [5] obtained the inequality $|b_{n}|\leq 2/(n+1)$ for $g\in\Sigma$ with $b_{k}=0$, $1\leq k< n/2$. For $g^{-1}$ being the inverse of *g*, Springer [17] showed that

$$ \vert B_{3} \vert \leq1 \quad \text{and} \quad \biggl\vert B_{3}+\frac{1}{2}B_{1}^{2} \biggr\vert \leq\frac{1}{2}, $$

and conjectured that

$$ \vert B_{2n-1} \vert \leq \frac{(2n-2)!}{n!(n-1)!} \quad (n=1,2,\dots). $$

In 1977, Kubota [9] proved that the Springer conjecture is true for $n=3,4,5$, and subsequently Schober [16] obtained sharp bounds for the coefficients $B_{2n-1}$ ($1\leq n \leq7$). Recently, Kapoor and Mishra [8] found coefficient estimates for inverses of meromorphic starlike functions of positive order *α* in Δ.

In the present investigation, certain subclasses of meromorphic bi-univalent functions are introduced and estimates for the coefficients $|b_{0}|$ and $|b_{1}|$ of functions in the newly introduced subclasses are obtained. These coefficient results are obtained by associating with the functions having positive real part. An analytic function *p* of the form $p(z)=1+c_{1}z+c_{2}z^{2}+\cdots$ is called a function with positive real part in $\mathbb{U}$ if $\Re p(z)>0$ for all $z\in\mathbb{U}$. The class of all functions with positive real part is denoted by $\mathcal{P}$. We need the following lemmas [13] to prove our main results.

### Lemma 1.1

*If* $\phi(z)\in\mathcal{P}$, *the class of functions analytic in* $\mathbb{U}$ *with positive real part*, *is given by*

$$ \phi(z)=1+c_{1}z+c_{2}z^{2}+c_{3}z^{3}+ \cdots\quad(z\in\mathbb{U}), $$

*then* $|c_{n}|\leq2$ *for each* $n\in\mathbb{N}$.

In 1972, the following univalence criterion was proved by Ozaki and Nunokawa [12].

### Lemma 1.2

*If for* $f\in\mathcal{A}$

$$ \biggl\vert \frac{z^{2}f'(z)}{[f(z)]^{2}}-1 \biggr\vert < 1\quad(z\in\mathbb{U}), $$

*then* *f* *is univalent in* $\mathbb{U}$ *and hence* $f\in\mathcal{S}$.

Also, let $\mathcal{T}(\mu)$ denote the class of functions $f\in\mathcal{A}$ such that

$$ \biggl\vert \frac{z^{2}f'(z)}{[f(z)]^{2}}-1 \biggr\vert < \mu\quad(z\in\mathbb{U}), $$

where *μ* is a real number with $0<\mu\leq1$ and $\mathcal{T}(1)=\mathcal{T}$. It is clear that $\mathcal{T}(\mu)\subset\mathcal{T}\subset\mathcal{S}$.

Further (see Kuroki et al. [10]), for $f\in\mathcal{T}(\mu)$ we have that:

$$ \Re \biggl(\frac{z^{2}f'(z)}{[f(z)]^{2}} \biggr)>1-\mu\quad(z\in\mathbb{U}). $$

## Main results

### Definition 2.1

A function $g\in\Sigma_{\sigma}$ given by (1.2) is said to be in the class $\mathcal{T}_{\Sigma_{\sigma}}^{\alpha}$ if the following conditions are satisfied:

2.1 $$ \begin{aligned} &\biggl\vert \arg \biggl(\frac{z^{2}g'(z)}{[g(z)]^{2}} \biggr) \biggr\vert < \frac{\alpha\pi}{2}\quad(z\in\Delta; 0< \alpha\leq1) \quad \text{and} \quad \\ &\biggl\vert \arg \biggl(\frac{w^{2}h'(w)}{[h(w)]^{2}} \biggr) \biggr\vert < \frac{\alpha\pi}{2}\quad(w\in\Delta; 0< \alpha\leq1) \end{aligned} $$

where the function *h* is an extension of $g^{-1}$ to Δ defined by (1.4).

### Definition 2.2

A function $g\in\Sigma_{\sigma}$ given by (1.2) is said to be in the class $\mathcal{T}_{\Sigma_{\sigma}}(\mu)$ if the following conditions are satisfied:

2.2 $$ \begin{aligned} &\Re \biggl(\frac{z^{2}g'(z)}{[g(z)]^{2}} \biggr)>1-\mu\quad(z\in\Delta; 0< \mu\leq1) \quad \text{and} \\ &\Re \biggl(\frac{w^{2}h'(w)}{[h(w)]^{2}} \biggr)>1-\mu\quad(w\in\Delta; 0< \mu\leq1) \end{aligned} $$

where the function *h* is an extension of $g^{-1}$ to Δ defined by (1.4).

### Theorem 2.1

*Let the function* *g*, *given by the series expansion* (1.2), *be in the function class* $\mathcal{T}_{\Sigma_{\sigma}}^{\alpha}$, $0<\alpha\leq1$. *Then*

2.3 $$\begin{aligned} &\vert b_{0} \vert \leq\sqrt{\frac{2}{3}} \alpha , \end{aligned}$$

2.4 $$\begin{aligned} &\vert b_{1} \vert \leq \textstyle\begin{cases} \frac{2}{3}\alpha, & 0< \alpha\leq\frac{\sqrt{2}}{2}, \\ \frac{2\sqrt{2}}{3}\alpha^{2}, & \frac{\sqrt{2}}{2}\leq\alpha\leq1. \end{cases}\displaystyle \end{aligned}$$

### Proof

It follows from (2.1) that

2.5 $$ \frac{z^{2}g'(z)}{[g(z)]^{2}}=\bigl[s(z)\bigr]^{\alpha} \quad \text{and} \quad \frac{w^{2}h'(w)}{[h(w)]^{2}}=\bigl[t(w)\bigr]^{\alpha}\quad (z\in\Delta) , $$

respectively, where $s(z)$ and $t(z)$ are functions with positive real part in Δ and have the forms

2.6 $$ s(z)=1+\frac{s_{1}}{z}+\frac{s_{2}}{z^{2}}+\cdots $$

and

2.7 $$ t(w)=1+\frac{t_{1}}{w}+\frac{t_{2}}{w^{2}}+\cdots, $$

respectively. Now, upon equating the coefficients in (2.5), we get

2.8 $$\begin{aligned} &{-}2b_{0}=\alpha s_{1} , \end{aligned}$$

2.9 $$\begin{aligned} &{-}3\bigl(b_{1}-b_{0}^{2}\bigr)= \alpha s_{2}+\frac{\alpha(\alpha-1)}{2}s_{1}^{2} , \end{aligned}$$

2.10 $$\begin{aligned} &2b_{0}=\alpha t_{1} , \end{aligned}$$

2.11 $$\begin{aligned} &3\bigl(b_{1}+b_{0}^{2}\bigr)= \alpha t_{2}+\frac{\alpha(\alpha-1)}{2}t_{1}^{2} , \end{aligned}$$

and, from (2.8) and (2.10), we find that

2.12 $$\begin{aligned} &s_{1}=-t_{1} , \end{aligned}$$

2.13 $$\begin{aligned} &8b_{0}^{2}=\alpha^{2} \bigl(s_{1}^{2}+t_{1}^{2}\bigr). \end{aligned}$$

Also from (2.9) and (2.11) we obtain

2.14 $$ 6b_{0}^{2}=\alpha(s_{2}+t_{2})+ \frac{\alpha(\alpha-1)}{2}\bigl(s_{1}^{2}+t_{1}^{2} \bigr). $$

Since $\mathfrak{R}(s(z))>0$ and $\mathfrak{R}(t(z))>0$ in Δ, the functions $s(1/z),t(1/z)\in\mathcal{P}$ and hence the coefficients $s_{k}$ and $t_{k}$ for each *k* satisfy the inequality in Lemma 1.1. Applying the triangle inequality, and then Lemma 1.1, in (2.13) and (2.14) gives us the desired estimates on $|b_{0}|$, as asserted in (2.3).

Next, in order to find the bound on the coefficient $|b_{1}|$, we subtract (2.11) from (2.9), and we thus get

2.15 $$ -6b_{1}=\alpha(s_{2}-t_{2}). $$

Hence

2.16 $$ |b_{1|}\leq\frac{2}{3}\alpha. $$

On the other hand, using (2.9) and (2.11) yields

2.17 $$\begin{aligned} &9\bigl(b_{1}-b_{0}^{2} \bigr)^{2}+9\bigl(b_{1}+b_{0}^{2} \bigr)^{2} \\ &\quad =\alpha^{2}\bigl(s_{2}^{2}+t_{2}^{2} \bigr)+\frac{\alpha^{2}(\alpha-1)^{2}}{4}\bigl(s_{1}^{4}+t_{1}^{4} \bigr)+\alpha^{2}(\alpha-1) \bigl(s_{1}^{2}s_{2}+t_{1}^{2}t_{2} \bigr). \end{aligned}$$

By using (2.13), we have from the above equality

2.18 $$\begin{aligned} 18b_{1}^{2}={}&\alpha^{2} \bigl(s_{2}^{2}+t_{2}^{2}\bigr)+ \frac{\alpha^{2}(\alpha-1)^{2}}{4}\bigl(s_{1}^{4}+t_{1}^{4} \bigr)+\alpha^{2}(\alpha-1) \bigl(s_{1}^{2}s_{2}+t_{1}^{2}t_{2} \bigr) \\ &{}-\frac{9\alpha^{4}(s_{1}^{2}+t_{1}^{2})^{2}}{32}. \end{aligned}$$

From Lemma 1.1 we obtain

$$ \vert b_{1} \vert ^{2}\leq\frac{13}{9} \alpha^{4}, $$

and therefore,

2.19 $$ \vert b_{1} \vert \leq\frac{\sqrt{13}}{3} \alpha^{2}. $$

Also, by using (2.14), we have from equality (2.17) that

$$\begin{aligned} 18b_{1}^{2} =&\alpha^{2}\bigl(s_{2}^{2}+t_{2}^{2} \bigr)+\frac{\alpha^{2}(\alpha-1)^{2}}{4}\bigl(s_{1}^{4}+t_{1}^{4} \bigr)+\alpha^{2}(\alpha-1) \bigl(s_{1}^{2}s_{2}+t_{1}^{2}t_{2} \bigr)\\ &{}-18 \biggl[\frac{\alpha(s_{2}+t_{2})}{6}+\frac{\alpha(\alpha-1)(s_{1}^{2}+s_{2}^{2})}{12} \biggr]^{2}. \end{aligned}$$

From Lemma 1.1 we obtain

2.20 $$ \vert b_{1} \vert \leq\frac{2\sqrt{2}}{3} \alpha^{2}. $$

Comparing (2.16), (2.19) and (2.20), we get the desired estimate on the coefficient $|b_{1}|$, as asserted in (2.4). □

### Theorem 2.2

*Let the function* *g*, *given by the series expansion* (1.2), *be in the function class* $\mathcal{T}_{\Sigma_{\sigma}}(\mu)$, $0<\mu\leq1$. *Then*

2.21 $$\begin{aligned} &\vert b_{0} \vert \leq\sqrt{\frac{2\mu}{3}} , \end{aligned}$$

2.22 $$\begin{aligned} & \vert b_{1} \vert \leq\frac{2\sqrt{2}}{3}\mu . \end{aligned}$$

### Proof

It follows from (2.2) that

2.23 $$\begin{aligned} &\frac{z^{2}g'(z)}{[g(z)]^{2}}=(1-\mu)+\mu s(z)\quad(z\in\Delta), \end{aligned}$$

2.24 $$\begin{aligned} &\frac{w^{2}h'(w)}{[h(w)]^{2}}=(1-\mu)+\mu t(z)\quad(z\in\Delta), \end{aligned}$$

respectively, where $s(z)$ and $t(w)$ are functions with positive real part in Δ and have the forms (2.6) and (2.7), respectively. Now, upon equating the coefficients in (2.23) and (2.24), we get

2.25 $$\begin{aligned} &{-}2b_{0}=\mu s_{1} , \end{aligned}$$

2.26 $$\begin{aligned} &{-}3\bigl(b_{1}-b_{0}^{2}\bigr)=\mu s_{2} , \end{aligned}$$

2.27 $$\begin{aligned} &2b_{0}=\mu t_{1} , \end{aligned}$$

2.28 $$\begin{aligned} &3\bigl(b_{1}+b_{0}^{2}\bigr)=\mu t_{2}. \end{aligned}$$

From (2.25) and (2.27) we obtain

2.29 $$\begin{aligned} &s_{1}=-t_{1} , \end{aligned}$$

2.30 $$\begin{aligned} &8b_{0}^{2}=\mu^{2} \bigl(s_{1}^{2}+t_{1}^{2}\bigr). \end{aligned}$$

Also from (2.26) and (2.28) we obtain

2.31 $$ 6b_{0}^{2}=\mu(s_{2}+t_{2}). $$

Since $\mathfrak{R}(s(z))>0$ and $\mathfrak{R}(t(z))>0$ in Δ, the functions $s(1/z),t(1/z)\in\mathcal{P}$ and hence the coefficients $s_{k}$ and $t_{k}$ for each *k* satisfy the inequality in Lemma 1.1. Therefore we find from (2.30) and (2.31) that

2.32 $$ \vert b_{0} \vert \leq\mu \quad \text{and} \quad \vert b_{0} \vert \leq\sqrt{\frac{2\mu }{3}} , $$

respectively. So we get the desired estimate on the coefficient $|b_{0}|$, as asserted in (2.21).

Next, in order to find the bound on the coefficient $|b_{1}|$, we subtract (2.28) from (2.26), and obtain

2.33 $$ 6b_{1}=\mu(t_{2}-s_{2}). $$

Hence

2.34 $$ \vert b_{1} \vert \leq\frac{2}{3}\mu. $$

On the other hand, using (2.26) and (2.28) yields

2.35 $$ -9\bigl(b_{1}-b_{0}^{2}\bigr) \bigl(b_{1}+b_{0}^{2}\bigr)=\mu^{2}s_{2}t_{2} , $$

or equivalently

2.36 $$ 9b_{1}^{2}=9b_{0}^{4}- \mu^{2}s_{2}t_{2}. $$

Upon substituting the value of $b_{0}^{2}$ from (2.30) and (2.31) into (2.36), respectively, it follows that

$$ \vert b_{1} \vert ^{2}\leq b_{0}^{4}- \frac{4}{9}\mu^{2} $$

and

2.37 $$ \vert b_{1} \vert \leq\frac{2\sqrt{2}}{3}\mu. $$

Comparing (2.34) and (2.37), we get the estimate desired on the coefficient $|b_{1}|$, as given in (2.22). □

## Conclusion

### Lemma 3.1

*If* $b_{0}=0$ *for the function* $g\in\Sigma$, *the series expansion* (1.2) *becomes*

$$ g^{-1}(w)=w-\frac{b_{1}}{w}-\frac{b_{2}}{w^{2}}-\frac{b_{1}^{2}+b_{3}}{w^{3}}+ \cdots . $$

*This series expansion was obtained by Schober* [16].

### Example 3.2

The function $g(z)=z+1/z$ is clearly a univalent meromorphic function. Direct calculation shows that

$$ g^{-1}(w)=\frac{w+\sqrt{w^{2}-4}}{2}. $$

This function has the series expansion given by

$$ g^{-1}(w)=w-\frac{1}{w}-\frac{1}{w^{3}}-\frac{2}{w^{5}}- \frac{5}{w^{7}}-\frac{14}{w^{9}}-\cdots . $$

### Corollary 3.1

*If* *g*, *given by* (1.2), *is in the class* $\mathcal{T}_{\Sigma_{\sigma}}(\mu)$, $0<\mu\leq1$, *and* $b_{0}=0$ *then*

$$ \vert b_{1} \vert \leq\frac{2}{3}\mu. $$

### Proof

Assume that the function $g(z)=z+\sum_{n=1}^{\infty}\frac{b_{n}}{z^{n}}\in\mathcal{T}_{\Sigma_{\sigma}}(\mu)$ where $0<\mu\leq1$. Since $b_{0}=0, \ s_{1}=t_{1}=0$, the result can be verified by a direct calculation of (2.36). □

### Corollary 3.2

*Let* $g\in\mathcal{T}_{\Sigma_{\sigma}}^{\alpha}$, *where* $0<\alpha\leq1$. *Then*

$$ \vert b_{1} \vert \leq\frac{2}{3}\alpha. $$

### Proof

Since the function $g(z)=z+\sum_{n=1}^{\infty}\frac{b_{n}}{z^{n}}\in\mathcal{T}_{\Sigma_{\sigma}}^{\alpha}$ where $0<\alpha\leq1$ and $b_{0}=0$, it follows that $s_{1}=t_{1}=0$. The result can now be seen by a direct calculation of (2.17). □
